# miRNA Regulatory Functions in Farm Animal Diseases, and Biomarker Potentials for Effective Therapies

**DOI:** 10.3390/ijms22063080

**Published:** 2021-03-17

**Authors:** Duy N. Do, Pier-Luc Dudemaine, Manisha Mathur, Prashanth Suravajhala, Xin Zhao, Eveline M. Ibeagha-Awemu

**Affiliations:** 1 Agriculture and Agri-Food Canada, Sherbrooke Research and Development Centre, Sherbrooke, QC J1M 0C8, Canada; dongocduy1@duytan.edu.vn (D.N.D.); Pier-Luc.dudemaine@canada.ca (P.-L.D.); 2 Institute of Research and Development, Duy Tan University, Danang 550000, Vietnam; 3 Advanced Milk Testing Research Laboratory, Postgraduate Institute of Veterinary Education and Research, Institute of Veterinary Education and Research (RAJUVAS), Jaipur 302001, Rajasthan, India; matmanisha@gmail.com; 4 Department of Biotechnology and Bioinformatics, Birla Institute of Scientific Research, Statue Circle, Jaipur 302001, Rajasthan, India; prash@bisr.res.in; 5 Department of Animal Science, McGill University, Ste-Anne-de Bellevue, QC H9X 3V9, Canada; xin.zhao@mcgill.ca

**Keywords:** livestock diseases, miRNAs, biomarkers, regulatory networks, mastitis, PRRSV, foot-and-mouth disease, Marek’s disease, RNAi therapy

## Abstract

MicroRNAs (miRNAs) are small endogenous RNAs that regulate gene expression post-transcriptionally by targeting either the 3′ untranslated or coding regions of genes. They have been reported to play key roles in a wide range of biological processes. The recent remarkable developments of transcriptomics technologies, especially next-generation sequencing technologies and advanced bioinformatics tools, allow more in-depth exploration of messenger RNAs (mRNAs) and non-coding RNAs (ncRNAs), including miRNAs. These technologies have offered great opportunities for a deeper exploration of miRNA involvement in farm animal diseases, as well as livestock productivity and welfare. In this review, we provide an overview of the current knowledge of miRNA roles in major farm animal diseases with a particular focus on diseases of economic importance. In addition, we discuss the steps and future perspectives of using miRNAs as biomarkers and molecular therapy for livestock disease management as well as the challenges and opportunities for understanding the regulatory mechanisms of miRNAs related to disease pathogenesis.

## 1. Introduction

MicroRNAs (miRNAs), defined as short non-coding RNA (ncRNA) molecules of about 22 nucleotides in length, regulate a variety of biological processes through the post-transcriptional regulation of gene expression. They control the expression of protein-coding genes and participate in the regulation of many cellular processes in animals. miRNAs regulate gene expression by inhibiting translation initiation or elongation, and inducing co-translational protein degradation and premature termination of translation [1,2]. In addition to this, they are also known to form miRNA–mRNA and miRNA–lncRNA pairs to influence gene regulation and biological activities [1].

From the identification of the first miRNA (lin-4) in 1993 [3], advances in next-generation (NGS) and third-generation sequencing (TGS) technologies in the last decade have heralded a new era and ability to identify various classes of small RNA molecules, including miRNA, in different biological samples at unprecedented speed [4]. The advantages offered by multiple sequencing platforms (e.g., Illumina, Ion Torrent and SOLiD) and bioinformatics data management capabilities support in-depth miRNA sequencing (miRNA-Seq) with the possibility to identify known and novel miRNAs [5,6], mutations [7], and their potential functions [8].

Along with NGS and TGS tools, bioinformatics tools for miRNA sequencing data analyses have progressed quickly from the development of pipelines for processing sequencing data to the inference of miRNA functions. The global processes and tools involved in miRNA discovery in NGS data have been summarized in recent reviews [4,9,10,11]. Following miRNA discovery, pathway analysis tools (e.g., miRNet [12] or miRPathDB 2.0 [13]) are used to predict their potential functions. Moreover, experimental, or wet lab approaches are used for the further functional validation of miRNA target genes and functions. Since the discovery of lin-4 [3], thousands of miRNAs have been identified in farm animal species and deposited in miRNA databases (Table 1).

Livestock diseases are responsible for huge economic losses to the livestock industry and cause important issues of animal welfare [14,15]. Moreover, many livestock diseases can be transmitted to humans with the potential to cause health issues and even death. The effective control of livestock diseases is a global challenge for the livestock industry requiring multiple layers of control and intervention [16,17,18]. Many livestock diseases, such as mastitis, paratuberculosis and bovine viral diarrhea (BVD) in cattle, porcine reproductive and respiratory syndrome (PRRS) and African swine fever (ASF) in pigs, and Newcastle disease and Avian influenza in poultry, require multidisciplinary or holistic approaches for effective management and control. Vaccination, therapeutic treatments, and eradication strategies are traditional and routine methods to combat diseases, while modern methods, such as genome editing and RNA interference (RNAi), can lead to the development of alternative strategies for combating disease outbreaks. However, instead of combating diseases, farmers can select animals based on the genetic resistance of health traits, which has been regarded as a sustainable method [14,15]. Together with genetic markers, targeting epigenetic markers and miRNAs have been regarded as a further strategy for combating livestock diseases [19,20,21,22,23].

The potential roles of miRNAs in farm animal diseases have been summarized in several reviews [24,25,26,27,28,29]. These reviews, however, only provided an overview of the changes in miRNA expression profiles during disease progression. In this review, we present an in-depth and up to date review of miRNA roles in the main farm animal diseases and argue for the potential use of miRNAs as biomarkers for animal disease management.

## 2. miRNA Biomarker Development and Potential Therapeutic Application in Livestock Production

The possibility and the practical aspects of using miRNAs as biomarkers have been intensively reviewed in many human diseases, such as cancers [30,31,32], rheumatic diseases [33], diabetes mellitus [34,35], and infectious diseases [36]. In farm animals, discovering biomarkers will be crucial for the management of disease [37].

A biomarker is a measurable indicator of a certain biological state, such as health and disease. Initial biomarkers used in human disease management were protein biomarkers but detecting new and enhanced protein biomarkers has proven to be an expensive and time consuming venture, mainly due to the low availability of clinically relevant proteins, their complex nature and the lack of accurate and repeatable detection methods [32]. Taylor [38] recently summarized the common qualifying factors of a biomarker: (1) easily accessible (i.e., discovered and measured easily and using minimal invasive procedures); (2) specific to the condition under investigation (specificity); (3) high sensitivity (easily and accurately detected, ideally before the appearance of clinical symptoms, and potentially vary according to disease stages or response to therapy); and (4) translatable from research/development to application. As compared to other nucleic acids, miRNAs are ideal biomarker candidates, as they are very stable under a wide range of conditions and can be extracted from a variety of liquid biospecimens (e.g., blood, urine, milk, and feces) and tissue samples. miRNAs are also highly specific to tissues and cell types, and their ability to delineate disease stages has been successfully used to differentiate cancer stages and to monitor responsiveness to therapy [39,40]. Specifically, a diagnostic role for miRNA with the ability to identify a disease or a prognostic role with the prospect of developing a specific disease phenotype has been demonstrated through meta-analyses of multiple cancer studies [41,42]. Moreover, miRNA signatures in bone marrow or blood are able to distinguish between Pediatric Acute Lymphoblastic Leukemia subtypes [43]. Moreover, miRNA biomarkers could enhance the specificity of metabolic or protein-based tests. For example, Ali Ahmed et al. showed recently that a combination of matrix metalloprotease protein-2 (MMP2) assay with miR-29a and miR-335 expression profiles demonstrated superior diagnostic detection of breast cancer compared to widely used tests, such as carcinoembryonic antigen (CEA) and cancer antigen 15-3 (CA 15-3) [44]. In farm animals, miRNA biomarker potentials for a range of diseases are reviewed below.

**Figure 1:**
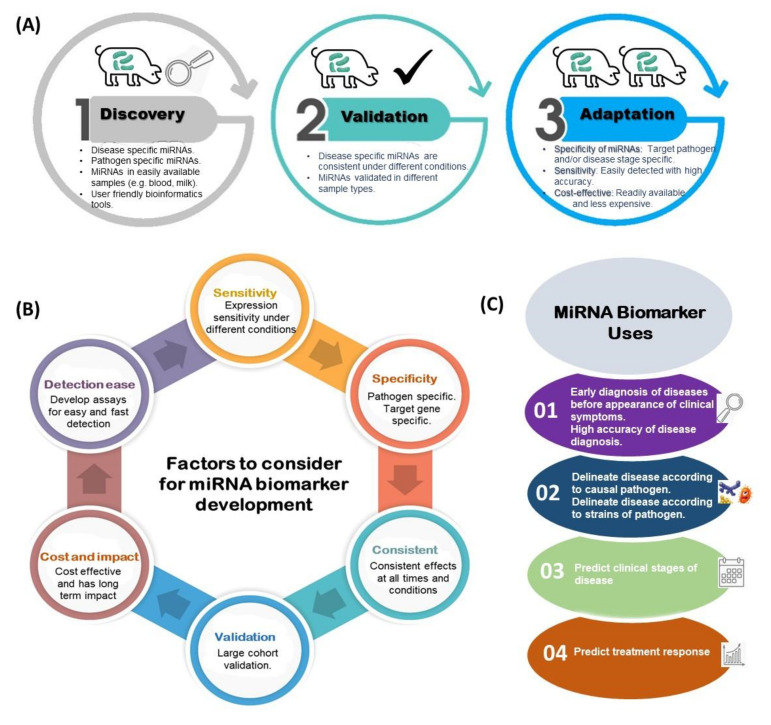
An overview of steps involved in the development of miRNA biomarkers for livestock disease management: (**A**) steps/stages to follow in miRNA biomarker development; (**B**) factors to consider in miRNA biomarker development; (**C**) potential application of miRNA biomarkers.

miRNA can be used as biomarkers, capable of serving diagnostic, prognostic, or therapeutic purposes, for the management of livestock diseases. The development of a biomarker generally requires three major steps. The first step includes (i) discovery: to identify the potential candidate markers for specific conditions; (ii) confirmation and validation: to test the identified markers in other populations or other samples; (iii) adaptation: development of suitable arrays or tests for the identified markers (Figure 1A). To date, most miRNA studies in animal diseases have focused on profiling miRNA changes (discovery stage). Although the functions of some miRNAs have been validated as summarized in the sections below, those experiments were mostly in vitro and may not reflect the actual underlying complex biological regulatory mechanisms. To the best of our knowledge, there are no available commercial miRNA biomarkers for use in livestock disease management.

Second, many factors must be considered, including specificity, expression sensitivity, validation in a large cohort to ensure its effectiveness, consistency of results, and impact on both cost (cost-effective) and time (long-term) (Figure 1B). Therefore, to qualify as an miRNA biomarker, expression specificity and consistency for a specific condition must be established in the initial population, and repeatability in other populations confirmed. Moreover, the long-term cost implications must be favorable to enable adoption by end users.

Third, a validated miRNA biomarker should find application in any of the following: diagnosis, prognosis, therapy, and for predicting the response to therapy (Figure 1C). For instance, different groups of miRNA biomarkers can be used for the management of Johne’s disease (JD) in livestock as follows: (i) for early diagnosis of JD infection, (ii) for classifying JD due to different strains of *Mycobacterium avium* subsp. *paratuberculosis* (MAP), and (iii) for predicting the stage of JD and (iv) as an effective treatment (therapeutic).

The potential application of miRNAs as biomarkers in livestock improvement is further strengthened by a plethora of recent reports, summarized in section three below, that have reported differential response patterns of miRNAs to different livestock diseases.

The development of miRNA biomarkers for livestock diseases, however, requires collective action and different stakeholders’ involvement. Researchers, veterinarians and farmers have the prerogative to identify the issues and the need for biomarkers for specific purposes. The experimental design and data analysis require both the statisticians and bioinformaticians to derive the best analytical tools to deliver robust results in the discovery and validation phases. A crucial link is required between academia (researchers) and industry to develop cost-effective and reliable tests and to select the best option for developing biomarkers. Finally, validated and reliable miRNA-based diagnostic kits and biomarkers for breeding for resistance will support livestock improved productivity. Therefore, successfully developed miRNA biomarkers might serve as new tools that could enhance current methods or lead to the development of new methods or therapies for managing farm animal diseases.

miRNA-based therapeutic approaches for the management of farm animal diseases can be through miRNA inhibition (diminish the expression of disease-induced miRNAs) or miRNA replacement (re-establish the expression of disease repressed miRNAs) [45]. Several approaches have been used for miRNA inhibition, such as antisense anti-miR oligonucleotides, locked nucleic acid anti-miRs, antagomiRs, and miRNA sponges [46]. Similarly, several small molecules, synthetic miRNA mimics, and DNA plasmids have been used in miRNA restoration therapy [47,48]. The success of miRNA therapies majorly depends on suitable, effective, and specific delivery systems [49,50]. Some highly efficient and specific miRNA delivery methods have been via exosome and nanoparticles [51,52]. miRNA therapies to cure diseases in livestock could be an attractive option to complement or replace current disease-management methods. However, understanding the specific functions and mechanisms of interaction between miRNA and other biomolecules in disease pathogenesis is the first step in the development of miRNA therapeutics for livestock diseases.

The advantages of miRNA biomarkers for disease management have been summarized recently [44] and include (1) use in early detection of disease, which is important to improve prognosis and limit the spread of disease pathogens; (2) use to improve pathogen detection at the first sign of appearance of disease symptoms; (3) use to detect the latent phase of infection, which is crucial to limiting the spread of pathogens and loss of income through lower productivity when undetected (e.g., the early and subclinical phases of JD); and (4) use to detect and manage regional differences in disease pathogenicity. miRNA biomarkers are also important, as they can be used to develop effective therapies for livestock diseases.

## 3. miRNA Roles in Farm Animal Diseases

In this section, we present reported changes in miRNA expression profiles during disease pathogenesis in farm animal species, including cattle, pig, chicken, sheep, and goat. We present the most recent findings and also focus on the most important economic diseases of livestock.

### 3.1. Potential Regulatory Roles of miRNAs in Cattle Diseases

Following the first miRNA expression study on bovine adipose, mammary gland, immune-related, and embryonic tissues in 2007 [53], over 870 studies have characterized about 1064 precursors and 1025 mature miRNAs, encoded on all 30 chromosomes, in the *Bos taurus* genome (Table 1). These studies demonstrate crucial regulatory roles of miRNAs in many biological processes in bovine, including mammary gland development and lactation (reviewed in [29]), bovine immunity (reviewed in [54]) and diseases (reviewed in [24]), and embryo development (reviewed in [55]). This section provides an update on miRNA signatures and potential roles in the major bovine infectious diseases, including mastitis, paratuberculosis, or JD and BVD. Important miRNAs for these diseases are listed in Table 2.

#### 3.1.1. miRNA and Mastitis

Mastitis, a common inflammatory disease of the mammary gland can develop into a clinical or subclinical type of infection depending on the causal pathogen [71]. Mastitis infection is caused by diverse pathogens including, but not limited to, *Escherichia coli*, *Streptococcus uberis, Streptococcus dysgalactiae*, *Bacillus spp,* and *Staphylococcus aureus* [72]. Dairy cow intramammary infections due to *S. aureus* have received much attention because of their major economic impact on dairy farms [73,74]. Therefore, more studies have investigated the role of miRNA functions in relation to this pathogen [56,57,60,62,75,76,77,78,79,80,81]. *E. coli* generally causes clinical mastitis, while most cases of *S. aureus* mastitis are chronic in nature. Investigating the miRNA expression profiles due to these pathogens following challenge of MAC-T cells (bovine mammary epithelial cell line) with heat-inactivated *S. aureus* or *E. coli* bacteria at 0, 6, 12, 24, and 48 h, Jin et al. [60], reported a pathogen directed miRNA expression pattern whereby four differentially expressed (DE) miRNAs (miR-2339, miR-499, miR-23a, and miR-99b) were unique to *S. aureus*, while 5 (miR-184, miR-24-3p, miR-148, miR-486, and let-7a-5p) were unique to *E. coli*. Interestingly, the authors also observed a slower initial response of miRNAs to *S. aureus* bacteria (only one DE miRNA reported after 6 h of infection) compared to *E. coli,* which initiated an earlier miRNA response (six DE miRNAs reported after 6 h of infection) [60]. Another report on pathogen specificity identified 108 DE miRNAs as specific to *E. coli*, while 82 DE miRNAs were specific to cows infected with *S. aureus* [62]. Meanwhile, several important miRNA candidate biomarkers (e.g., mi-223, miR-1246, miR-142-5p, miR-23a, miR-31, miR-23b-3p, miR-26a, and miR-145) have been associated with *S. aureus* and/or *E. coli* mastitis in Chinese Holstein cows [76]. Moreover, Ma et al. [81] recently identified miR-378 and miR-185 as candidate biomarkers of milk infected with *S. aureus*. These studies and other lines of evidence indicate that miRNAs regulate the host response to *S. aureus* via different target genes and pathways. For example, miR-223 regulation of *S. aureus* resistance is via the PI3K/AKT/NF-κB pathway [80]; miR-145 modulation is through pathways related to immune cytokines [78]; and miR-15a regulation is through the inhibition of Interleukin-1 Receptor-Associated Kinase 2 (*IRAK2*) gene expression (Chen et al.) [79].

*S. uberis* is among the most prevalent mastitis-causing pathogens throughout Europe and North America [82]. Using real-time quantitative PCR to examine the expression of 14 miRNAs in bovine mammary epithelial cells (BMECs) challenged with *S. uberis,* Naeem et al. [57] indicated possible roles of miR-181a in intramammary infections via its regulatory function on Fc-gammaR-mediated phagocytosis, toll-like receptor signaling, and antigen processing and presentation pathways, while using a next generation sequencing approach, Lawless et al. identified 21 miRNAs as significantly DE post-infection as well as enrichment in pathways related to innate immunity [56]. Ngo et al. [58] profiled circulating miRNAs in cows’ milk with naturally occurring mastitis due to different causative agents and identified 26 miRNAs as generic indicators of clinical mastitis, and suggested seven of them (miR-27b, miR-152, miR-194, miR-200b, miR-222, miR-379, and miR-18397) as early mastitis indicators. The authors identified 27 miRNAs unique to *S. uberis* mastitis with an emphasis on miR-320a and miR-320b due to their roles in the modulation of trained immune activity [58].

Compared to *S. uberis* and *S. aureus*, less attention has been given to miRNA changes during *E. coli* and *S. agalactiae* mastitis. Pu et al. [61] identified 35 DE miRNAs in mammary gland tissues from cows with *S. agalactiae-*type mastitis, with regulatory roles in several immune response and signal transduction pathways, such as the RIG-I-like receptor signaling pathway, cytosolic DNA sensing pathway, and Notch signal pathway. Analyzing miRNA expression profiles from peripheral blood, Li et al. [59] reported the involvement of several miRNAs (miR-25, miR30e-5p, miR-342, miR-191, miR-399b, miR-451, and miR-486) in biological processes involved in mastitis infection, such as the involvement of miR-25 in the development of the immune system through targeting of Krüppel-like factor 4 (*KLF4*) gene [59]. Meanwhile, Chen et al. [83] suggested miR-16 and miR-223 as new markers for dairy cow mastitis diagnosis. Interestingly, Lai et al. [63] identified five significantly upregulated miRNAs (miR-21, miR-146a, miR-155, miR-222, and miR-383) with the potential to effectively differentiate between California mastitis test-positive milk and non-infected milk. In another study, miR-144-5p and miR-130b-5p were significantly downregulated and upregulated, respectively, in mammary gland tissues infected with mastitis compared to healthy tissues [84].

#### 3.1.2. miRNA and Johne’s Disease

*Mycobacterium avium* subsp. *paratuberculosis* (MAP) is the causative agent of JD in cattle, sheep, goats, and other domestic and wild animals [85,86]. Johne’s disease imposes a substantial economic burden on the dairy industry [85,87], and it is prevalent worldwide [88]. MAP and JD have attracted attention because of their speculated connection to human Crohn’s disease [89]. Current JD control strategies are hampered by the lack of accurate and reliable diagnostic tests [90]. Meanwhile, several studies have indicated the potential of miRNAs to serve as diagnostic and prognostic tools of MAP. For example, Malvisi et al. [65] identified seven upregulated (miR-19b, miR-19b-2, miR-1271, miR-100, miR-301a, miR-32, and one novel miRNA) and two downregulated (miR-6517 and miR-7857) miRNAs in the blood of MAP-positive animals compared with unexposed animals. Studying the ileum, a target tissue for MAP infection, Liang et al. [66] reported 14  DE miRNAs as well as the potential role of miR-196b in the proliferation of endothelial cells, the role of miR-146b in bacteria recognition, and involvement of miR-146b in the regulation of the inflammatory response when comparing infected and control ileum tissues from calves. Recently, Gupta et al. [67] developed a prediction model using a combination of four miRNAs (miR-1976, miR-873-3p, miR-520f- 3p, and miR-126-3p), which distinguished animals moderately and severely infected with JD from healthy animals. Ileum and ileal lymph nodes are important tissues during MAP infection, as they are the sites of MAP and host immune cell interaction, and where the host initiates immune responses to the pathogen. Wang et al. [91,92] reported the involvement of a different set of miRNAs in the regulation of MAP response in the ileum and ileal lymph as well as the possible involvement of miR-100, miR-330, and miR-2447 in the Th17 cell differentiation pathway during MAP infection in the ileal lymph node [91] or association of miR-370 and miR-383 with lipid metabolism in this tissue [92]. Moreover, Shaughnessy et al. demonstrated the utility of a number of miRNAs in bovine feces in differentiating healthy animals from those with late-stage JD, thereby providing potential biomarkers for MAP infection and disease progression [68].

#### 3.1.3. miRNA and Other Cattle Diseases

miRNA expression is also changed during the progression of some other notable infectious diseases of bovine, including BVD, foot and mouth disease (FMD), and tuberculosis ([69,93,94]). Like JD, the symptoms of BVD virus (BVDV) infections in cows are subclinical and difficult to detect. Only one study has investigated the miRNA profiles of colostrum infected with BVDV from five neonate Holstein calves inoculated with BVDV at different time points: before infection (day 0) and at 4, 9, and 16 days post-challenge and reported two DE miRNAs (miR-423-5p and miR-151-3p) between BVDV challenged and control groups across time points [69]. However, both miRNAs demonstrated inconsistent expression patterns, whereby miR-423-5p increased until day 4 post-challenge and decreased to the control level by day 16 post-BVDV exposure. At the same time, miR-151-3p remained similar to the control level until day 9 before increasing in BVDV challenged animals compared to control animals on day 16 [69]. Thus, more studies are required to identify miRNA roles in BVDV and miRNA biomarkers of BVDV. FMD is a highly contagious disease of domestic and wild cloven-hoofed animals [93], and its outbreaks incur enormous economic, political, and social ramifications. A recent study has suggested potential roles of miR-17-5p in acute FMD infection, miR-31 in FMD virus (FMDV) persistence, and miR-1281 in both acute and persistent infection [70]. Profiling miRNA expression in alveolar macrophages isolated from the lung of bovine infected with *Mycobacterium bovis*, the causative agent of bovine tuberculosis, Vegh et al. identified different sets of DE miRNAs, with crucial roles in the tightly controlled balance between pathogen survival strategies and the host immune response [64]. Among them, the authors validated IL-1 receptor-associated kinase 1 (*IRAK1*) and TGF-β receptor 2 (*TGFBR2*) as important target genes of miR-146. In addition, Want et al. [95] indicated that miRNA-199a plays important roles in *M. bovis* infection via the inhibition of cellular autography and downregulation of IFN-β expression, while Iannaccone et al. [94] identified miR-146a as a potential biomarker for the rapid diagnosis of *M. bovis* infection.

### 3.2. Potential Regulatory Roles of miRNA in Pig Diseases

The first set of porcine miRNAs identified through sequence homology search with known human miRNAs belonged to the miR17-92 cluster and included miR-17, miR-18a, miR-19a, miR-20a, miR-19b, and miR-92a [96]. Since then, a total of 408 precursors and 457 mature porcine miRNAs have been reported and deposited in miRNA databases (Table 1). Studies on miRNA functions have focused on specific diseases or pathogens, mostly using in vivo challenge experiments. A number of important miRNAs for pig diseases are listed in Table 3.

#### 3.2.1. miRNA and Porcine Reproductive and Respiratory Syndrome Virus Infection

Porcine reproductive and respiratory syndrome virus (PRRSV) is among the most important viral pathogens in the swine industry [122]. The first miRNA study in relation to PRRSV infection used Illumina deep sequencing to construct small RNA expression profiles from porcine alveolar macrophages infected with cultured PRRSV [97]. The authors detected 40 DE miRNAs within the first 48 h post-infection (hpi), while the expression of six miRNAs (miR-30a-3p, miR-132, miR-27b, miR-29b, miR-146a, and miR-9-2) was altered at more than one time point. Furthermore, a miR-147 mimic experiment indicated that PRRSV replication was negatively impacted by the high expression level of miR-147 [97]. Several subsequent studies highlighted the roles of miR-181(*ORF4)* [98], miR-125b (*NF-κB*) [99], miR-23 (*IFN-*α) [100], miR-26 family (*IFN-α, MX1* and *ISG15*), miRNA-30c (*IFN-α*) [104], miR-22 (*HMOX1*) [105], miR-373 (*NFIA*, *NFIB*, *IRAK1, IRF1*) [106], miR-10a-5p (*SRP14*) [108], and miR-c89 (*RXRB*) [123], and their target genes in PRRSV infection. For instance, miR-181 could directly impair PRRSV infection in vitro through specific binding to a highly (over 96%) conserved region in the downstream region of *ORF4* (open reading frame 4) of the viral genomic RNA [98]. Furthermore, miR-181 can downregulate the PRRSV receptor *CD163* in blood monocytes and porcine alveolar macrophages [98]. Moreover, the downregulation of *CD163* led to the inhibition of PRRSV entry into porcine alveolar macrophages and subsequent suppression of PRRSV infection [98]. Meanwhile, Zhou et al. [101] observed that miR-145 was strongly induced by PRRSV infection, whereas miR-127 expression was significantly reduced at all infection time points in MARC-145 cells challenged with PRRSV. Results of miRNA expression profiles of lung tissues from Tongcheng or Landrace pigs infected with a highly pathogenic PRRSV strain indicated that miR-183, miR-219, miR-28-3p, and miR-143-3p were upregulated significantly at 3, 5, and 7 days post-infection (dpi) in both breeds [102]. The potential functions of the target genes of DE miRNAs have been reported, whereby miR-22 promotes PRRSV replication by targeting the Heme oxygenase-1 (*HMOX1*) gene of the host cells [105]; miR-373 by inhibiting the production of beta interferon (IFN-β) via targeting nuclear factor IA (*NFIA*), *NFIB*, interleukin-1 receptor-associated kinase 1 (*IRAK1*), *IRAK4*, and interferon regulatory factor 1 (*IRF1*) [106]; miR-10a-5p inhibition by targeting the host factor signal recognition particle 14 (*SRP14*) [108]; and miR-c89 inhibition of PRRSV replication by regulating the expression of the host factor porcine retinoid X receptor β (*RXRB*) [123]. Comparing the miRNA expression profiles of alveolar macrophages of indigenous Chinese Tongcheng pigs infected with PRRSV, Zhou et al. identified 23 upregulated and 25 downregulated miRNAs as well as the potential epigenetic roles of miRNAs (miR-19a-3p, miR-29a-3p, miR-29c-3p, and miR-342-3p) through their downregulation of methylation-related genes during PRRSV infection [107]. Results of an examination of the effect of PRRSV infection on the expression of 89 miRNAs yielded candidates with potential anti- and pro-viral functions, such as the predicted ability of miR-125b to limit PRRSV viral levels and miR-145-5p to cause alternative macrophage priming [109]. Therefore, highly pathogenic type 2 strain-PRRSV infection affects host homeostasis through changes in miRNA expression and influence on host immune, metabolic, and structural pathways [109].

#### 3.2.2. miRNA and Swine Influenza Infection

Influenza is a zoonotic viral disease that represents a health and economic threat to humans and animals worldwide [124]. In mammals, influenza viruses replicate mainly in the respiratory tract, usually accompanied by clinical signs, whereas in avian species, the major replication site is the intestinal tract without clinical symptoms [125]. Several studies with different approaches have investigated the implication of miRNAs in swine influenza infection [104,105]. Using computational procedures, initial research identified 36 pig miRNAs with putative target genes in swine influenza viral sequences isolated in a period of 38 years, which indicated that putative target genes and host miRNA (miR-124a, miR-136, and miR-145) interactions were maintained almost throughout virus evolution [110]. Based on miRNA expression profiling, several authors suggested that DE miRNAs regulate genes involved in innate immunity [111] or apoptosis and cell cycle regulation [120] in pigs infected with influenza virus. In another study, Brogaard et al. suggested five miRNAs (miR-15a, miR-18a, miR-21, miR-29b, and miR-590-3p) as potential modulators of viral pathogen recognition and apoptosis in the lung tissues of pigs on day 3 following challenge with influenza A virus H1N2 [121]. Moreover, Zhang et al. [126] observed that swine influenza virus H1N1/2009 infection could modulate the expression of host miRNAs (miR-204 and miR-4331) to facilitate its replication in the host.

#### 3.2.3. miRNA and Other Pig Diseases

Salmonella species infect many vertebrate species, including pigs. Pigs colonized with *Salmonella enterica serovar Typhimurium* are usually asymptomatic, making their detection in carrier pigs difficult. Variable fecal shedding of Salmonella is an important cause of foodborne illnesses [127], and effective control of Salmonella-related infections is of public health importance. Studying the role of miRNAs in Salmonella disease pathogenesis, Huang et al. demonstrated the decreased expression of miR-155 in pigs with persistent Salmonella shedding (PS) [112], while Hoeke et al. [113] showed that miR-29a regulates intestinal epithelial cell proliferation by targeting caveolin-2 (*CAV2*). Li et al. analyzed the miRNA expression profiles in porcine intestines infected with Lawsonia intracellularis and found that associated DE miRNAs could target genes involved in pathways related to the immune response, amino acid metabolism, and cell communication/growth/motility [114]. Huang et al. [128] compared the whole blood miRNA transcriptomes of pigs (Duroc × Landrace × Yorkshire) at 2 days post-inoculation and before Salmonella infection and identified 29 DE miRNAs, including miR-146a-5p, miR-125a, and miR-129a-5p, with roles in Salmonella infection and immunology signaling pathways. Following validation by real time quantitative PCR, the authors concluded that miR-146a-5p in peripheral blood could significantly increase the fecal bacterial load [128].

*E. coli* F18 is among the main causal pathogens of post-weaning diarrhea in piglets. Ye et al. [115] reported 58 DE miRNAs in *E. coli* F18-sensitive pigs and identified miR-143, let-7f, miR-30e, miR-148a, miR-148b, miR-181a, miR-192, miR-27b, miR-15b, miR-21, miR-215, and miR-152 as potential miRNA markers for *E. coli* F18. Using the Meishan piglet as a model animal to test their susceptibility to *E. coli* F18, Wu et al. [116] identified miR-196b, miR-499-5p, and miR-218-3p as candidate miRNAs involved in *E. coli* F18 infection. Furthermore, the authors noted that miR-218-3p might regulate Discs Large MAGUK Scaffold Protein 5 (*DLG5*) gene in *E. coli* F18-resistant pigs. *Clostridium perfringens* (*C. perfringens*) type C causes piglet diarrhea with serious economic consequences to the swine industry. Wang et al. [129] compared the ileum miRNA expression profiles of control pigs with pigs susceptible or resistant to *C. perfringens* and identified Nuclear Factor of Activated T Cells 4 (*NFATC4*), ETS-Like Gene 1 (*ELK*1)/Heat Shock Protein Family A (*Hsp70*)/Member 2 (*HSPA2*)/Interleukin 7 Receptor (*IL7R*), and Cardiotrophin Like Cytokine Factor 1 (*CLCF1*) as target genes of miR-7134-5p, miR-500, and miR-92b-3p, respectively, in response to *C. perfringen* infection.

Furthermore, let-7d-3p was suggested as a candidate for porcine whipworm (*Trichuris suis*) infection [117], and miR-664-5p, miR-451, and miR-15a as candidate miRNAs involved in pig response to *Actinobacillus pleuropneumoniae* infection [118]. Núñez-Hernández et al. [120] identified 12 DE miRNAs, seven dpi and three dpi (including four upregulated miRNAs: miR-451, miR-145-5p, miR-181a, and miR-122, and eight down regulated miRNAs: miR-92a, miR-23a, miR-92b-3p, miR-126-5p, miR-126-3p, miR-30d, miR-23b, and miR-92c) in both spleen and submandibular lymph node tissues from pigs experimentally infected with a virulent (E75) African swine fever virus. Zhang et al. [130] highlighted that miR-122 represses the protein expression and viral DNA replication of Porcine circovirus type 2 (PCV2) by down-regulating the expression of nuclear factor of activated T-cells 5 (*NFAT5*) and aminopeptidase puromycin sensitive (*NPEPPS*) in PK15 cells. Moreover, Li et al. [131] analyzed the expression profiles of miRNAs in PCV2-infected and non-infected cells and reported 44 DE miRNAs with potential roles in cellular inflammatory responses and cytokine dysfunction.

### 3.3. Potential Regulatory Roles of miRNAs in Poultry Diseases

The first study on chicken miRNAs identified 25 miRNAs from chicken embryos and adult chicken tissues (cerebrum, cerebellum, heart, lung, liver, kidney, and spleen) through small RNA cloning and sequencing [132]. Since then, many studies have explored miRNA expression in relation to both production and disease traits in chickens. About 882 precursors and 1232 mature miRNAs have been reported for chickens (Table 1). Potential miRNA biomarkers of some poultry diseases, including Marek’s disease, avian leukosis virus, and infectious bursal disease virus, are listed in Table 4.

#### 3.3.1. miRNA and Marek’s Disease Virus Infection

Marek’s disease, a highly contagious viral neoplastic disease caused by infection of *Gallid herpes virus* 2 (GaHV-2) or Marek’s disease virus (MDV), has remained a major concern in the poultry industry owing to the continual emergence of new virulent strains [157]. Several in vitro approaches have been employed to unravel the roles played by host-encoded miRNAs in different scenarios of MDV infection [133,135,136,137,157,158,159,160,161,162]. Using white leghorn chicken experimentally inoculated with the oncogenic RB-1B strain as a model to investigate the connection between chicken miRNA response and the oncogenic nature of MDV, Stik et al. [158] reported the upregulation of miR-21 in chicken inoculated with RB-1B strain as compared to chicken vaccinated with a non-oncogenic strain CVI988. In a similar experiment, Li et al. [135] showed that miR-26a was downregulated in chicken spleens infected with MDV during different phases of tumor formation, while Han et al. [136] indicated that miR-103-3p was downregulated in tumor samples from the spleen and liver of infected chickens. Zhao et al. [137] observed that miR-219b promoted cell apoptosis via the regulation of the expression of genes in the apoptosis pathways during MDV infection. Moreover, Heidari et al. [138] indicated that DE miRNAs are involved in many reactome immune-related pathways, such as cytokine signaling, innate and adaptive immune systems, Toll-like receptors, and interleukin pathways.

#### 3.3.2. miRNA and Avian Leukosis Virus Infection

Avian leukosis virus (ALV) belongs to the genus *Alpharetrovirus* of the *Retroviridae* family. This virus can induce tumors in avian hosts, including B-cell lymphoma, hemangioma, and myelocytoma [163]. In a study examining the miRNA-mediated control of avian leukosis virus subgroup J (ALV-J) infection, Li et al. [139] proposed that seven upregulated miRNAs (miR-221, miR-222, miR-1456, miR-1704, miR-1777, miR-1790, and miR-2127) identified in the liver of 10-week-old chickens infected with ALV-J might play a tumorigenic role, whereas five down regulated miRNAs (let-7b, let-7i, miR-125b, miR-375, and miR-458) might have associations with loss of tumor-suppressive functions. Li et al. [140] observed that the overexpression of miRNA-375 repressed yes-associated protein 1 (*YAP1*), cyclin E (*CCNE1*), and *Drosophila* inhibitor of apoptosis protein 1 (*DIAP1*) genes, and consequently decreased DF-1 cell proliferation. Furthermore, miR-221 was found to act as a tumorigenic agent by targeting the B-cell lymphoma 2 (*BCL-2*) modifying factor in liver tumors from chickens infected with ALV-J [142]. In the spleen of chickens infected with ALV-J, miR-23 was found to suppress interferon regulatory factor 1 (*IRF1*), thus allowing enhanced virus replication, while miR-34b-5p can suppress the melanoma differentiation-associated gene 5 (*MDA5*) signaling pathway to promote ALV-J-infected cell proliferation and ALV-J replication [144]. Moreover, Ji et al. [145] reported temporal changes of miRNA let-7b and let-7i expression in chickens challenged with subgroup ALV-J. Additionally, Zhou et al. [146] revealed that some miRNAs (miR-184-3p, miR-146a-3p, miR-146a-5p, miR-3538, and miR-155) participated in virus–vector interaction, oxidative phosphorylation, energy metabolism, and cell growth in CEF cells infected with ALV-J.

#### 3.3.3. miRNA and Other Chicken Diseases

Bursal disease, caused by infectious bursal disease virus (IBDV), is a highly contagious disease that predominantly affects the bursa of Fabricius in birds [164,165]. IBDV targets the host immune system by destroying B lymphocytes, attracting T cells, and activating macrophages [165]. In poultry, vaccination has contributed to the overall reduction of disease burden [166]; however, a comprehensive understanding of the complexity of virus and host interaction is limited [165]. The roles of miRNAs in the regulation of IBDV infection have been the focus of many investigations. Initially, Shen [167] reported that recombinant avian adeno-associated virus (AAAV)-delivered VP1- and VP2-specific miRNAs can inhibit the replication of IBDV efficiently in transducing 8-day-old specific pathogen-free chicken embryos. Two other studies reported the important roles of miR-9a and miR-2127 in IBDV infections. Ouyang et al. [147] found that miR-9 was induced 2, 4, 12, and 24 h after infection with IBDV and that miR-9 can promote IBDV replication by repressing the production of type I IFN. In a subsequent experiment, Ouyang et al. [148] provided evidence that miR-2127 function in IBDV infection is via the downregulation of CHP53 mRNA translation and attenuation of CHP53-mediated antiviral innate immune response against IBDV. More recently, Fu et al. [149] reported that miR-130b suppresses IBDV replication via directly targeting the viral genome and cellular Suppressor Of Cytokine Signaling 5 (*SOCS5*) gene.

Avian influenza, caused by avian influenza viruses (AIVs), is an important disease for many bird species. Wild waterfowls or aquatic birds are the natural reservoir hosts of all influenza A subtypes [168], except for two novel IAV subtypes, H17N10 and H18N11, in bats [169]. The first study on miRNA gene expression in chickens infected with AIV using a deep sequencing approach was performed by Wang et al. [150], who reported 73 and 36 DE miRNAs between chicken lungs and tracheae infected and not with low pathogenic H5N3, respectively, 4 dpi. Some of the DE miRNAs, such as miR-146, miR-15, and miR-21, function in immune-related signal pathways in mammals. In broiler chicken, Wang et al. [151] suggested miR-34a, miR-122-1, miR-122-2, miR-146a, miR-155, miR-206, miR-1719, miR-1594, miR-1599, and miR-451 as strong candidate miRNAs and *MX1, IL-8, IRF-7*, and *TNFRS19* as strong candidate genes involved in the regulation of host response to AIV infection. Examining chicken embryo fibroblasts infected and not with avian influenza virus H9N2, Peng et al. [152] identified 48 DE miRNAs (e.g., miR-146c, miR-181a, miR-181b, miR-30b, miR-30c, miR-30e, and miR-455), which were predicted to target immune response-related genes. When performing miRNA expression profiling in the spleen, thymus, and bursa in chicken and duck, Li et al. [170] reported divergent changes in the miRNA expression upon H5N1 infection in both breeds and suggested that miRNAs can account for the level of susceptibility upon H5N1 infection. Recently, O’Dowd et al. [171] indicated that miR-146a, miR-146b, miR-205a, miR205b, and miR-449 can be used as miRNA-based antiviral agents or vaccine adjuvants in alternative strategies for the control of AIV in chickens.

Chronic respiratory diseases caused by *Mycoplasma gallisepticum* have severe consequences in the poultry industry. Several potentially important miRNAs for this disease were recently suggested and include miR-8, miR-499 and miR-17 families [153], miR-99a (targets *SMARCA5*) [154], miR-101-3p [155], and miR-19a [156]. For instance, miR-19a suppresses the expression of *ZMYND11* in chicken embryonic lungs infected with *Mycoplasma gallisepticum* and DF-1 cells and activate the NF--κB signaling pathway and promote pro-inflammatory cytokine expression, cell cycle progression, and cell proliferation to defend against *Mycoplasma gallisepticum* infection [156].

### 3.4. Potential Regulatory Roles of miRNAs in Small Ruminant Diseases

Small ruminants, including sheep and goats, are an important source of meat, milk, and wool throughout the world. miRNA studies in small ruminants have focused on muscle [172,173,174], embryo/ovary [175,176,177,178,179], mammary gland development [180,181,182,183], milk-related phenotypes (yield and composition) [183,184,185], and hair/skin-related phenotypes [186,187,188]. An initial study on miRNA in small ruminants performed by Wenguang et al. [187] used microarray analysis to characterize the expression of 159 miRNAs in skin samples from the body and ear of goats and sheep and identified 105 miRNAs conserved between the two species with significant roles in hair follicle differentiation. Subsequent studies using high-throughput sequencing techniques identified 106 precursors and 153 mature miRNAs in sheep and 267 precursors and 436 mature miRNAs in goats (Table 1). miRNAs with important roles in sheep and goat diseases are shown in Table 5.

The functions of miRNAs have been reported for several small ruminant diseases, such as *Cystic echinococcosis* infection [189], an epithelial tumor induced in goats and sheep by enzootic nasal tumor virus (ENTV) [190], bluetongue virus [191], Peste des petits ruminants (PPR) infection [192], and prion disease [193]. Small ruminants are highly susceptible to *Cystic echinococcosis*, a chronic zoonotic infection caused by infection with the larval stage of the cestode, *Echinococcus granulosus*. Jiang et al. [189] profiled the miRNA expression in intestinal tissues of sheep with resistant and non-resistant Major Histocompatibility Complex (MHC) haplotypes after peroral infection with *E. granulosus* eggs and highlighted miR-21-3p, miR-542-5p, miR-671, miR-134-5p, miR-26b, and miR-27a as NF-κB pathway-responsive miRNAs during *E. granulosus* infection. miRNAs also play important roles in enzootic nasal adenocarcinoma, an epithelial tumor induced in goats and sheep by enzootic nasal tumor virus [190]. Wang et al. [190] identified 116 DE miRNAs in the tumor and para-carcinoma nasal tissues of Nanjing yellow goats with enzootic nasal adenocarcinoma and showed the involvement of the predicted target genes in cell proliferation, signal transduction, and other processes associated with cancer.

In an effort to explore the mechanisms of bluetongue virus infection, Du et al. [191] identified 25 known and 240 novel DE miRNA candidates in primary sheep testicular cells infected with bluetongue virus as well as the significant enrichment of target genes in MAPK, PI3K-Akt, endocytosis, Hippo, NF-κB viral carcinogenesis, FoxO, and JAK-STAT signaling pathways. Peste des petits ruminants (PPR) is a highly contagious viral disease characterized by fever, sore mouth, conjunctivitis, gastroenteritis, and pneumonia, and primarily affects goats and sheep. Recently, Pandey et al. [192] found that miR-21-3p, miR-320a, and miR-363 might act cooperatively to enhance viral pathogenesis in the lung and spleen of sheep by downregulating several immune response genes in lung and spleen tissues of sheep infected with PPR virus (PPRV). In goats, Qui et al. [196] identified 316 DE miRNAs between the control and infected peripheral blood mononuclear cells with PPR virus. The authors suggested various biological processes and pathways in which DE miRNAs might participate, such as immune functions (miR-150, miR-146, and let-7) or apoptosis (miR-671-5p and miR-182). In two follow-up studies, the authors validated the immunological roles of miR-1 via targeting Tumor Necrosis Factor-Like Weak (TWEAK) and miR-218 via targeting Signaling Lymphocyte Activation Molecular (SLAM) genes, respectively [197,198]. To identify potential miRNA biomarkers for small ruminant prion diseases, Sanz Rubio et al. [193] analyzed ten potential candidate miRNAs from circulating blood plasma of naturally infected scrapie sheep by quantitative reverse transcription PCR and identified miR-342-3p and miR-21-5p as circulating biomarkers of prion disease. Yang et al. [194] reported miR-150, miR-370-3p, and miR-411b-3p as important for PPR vaccine virus responses in both peripheral blood lymphocyte and primary testicular cells from sheep using time course experiments. Examining seronegative and infected sheep with Small Ruminant Lentiviruses, Bilbao-Arribas et al. [195] reported 52 DE miRNAs and suggested miR-21 as a potential biomarker for the severity of lung lesions or a therapeutic target.

### 3.5. Important miRNA Biomarkers in Livestock Diseases

**Figure 2:**
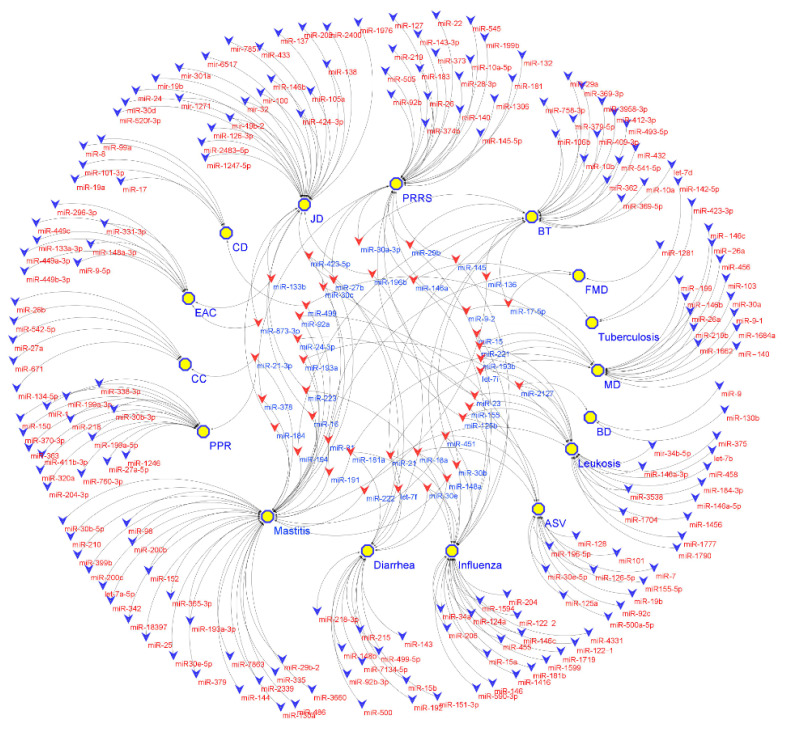
miRNAs in livestock diseases. Each V node represents an miRNA (blue V nodes (outer circle) represent miRNAs associated to one disease, and red V nodes (inner circle) present miRNAs associated to more than one disease) and each yellow hexagon node represents a livestock disease. FDM: foot and mouth disease; PRRS: porcine reproductive and respiratory syndrome; ASV: African swine fever virus; MD: Marek’s disease; BD: bursal disease; CD: chronic respiratory diseases; PPR: Peste des petits ruminants; CC: Cystic echinococcosis; EAC: enzootic nasal adenocarcinoma; BT: bluetongue; JD: Johne’s Disease.

Even though each disease reviewed has its own specific mechanisms, a common feature was the roles of associated miRNAs in the host immune response. miRNAs are essential players in innate immune and inflammatory responses [199,200]. Several notable miRNAs with roles in the innate immune regulations (reviewed in sections above) were associated with three or more livestock diseases, including miR-146, miR-223, and miR-21, etc. (Figure 2).

In humans, miR-146 is known as a key modulator of the immune response in cancers [201]. MiR-146 was associated with most diseases analyzed, such as bovine mastitis, tuberculosis, JD, swine influenza disease, PPRSV, and Avian influenza (Table 2, Table 3 and Table 4 and Figure 2). MiR-223 is an important miRNA in infection and inflammation [202,203], and it also plays important roles in bovine mastitis and swine influenza (Table 2 and Table 3, Figure 2). MiR-21 is an miRNA with multiple roles in human diseases [204], and it is also involved in multiple diseases in different livestock species (Table 2, Table 3, Table 4 and Table 5 and Figure 2). For instance, miR-21 modulates the expression of both pro- and anti-inflammatory cytokine responses against influenza A (H1N2) infection in pigs [121] or plays important roles in lymphocyte development and modulation in the lungs of chickens infected with AIV [150]. In addition to the common roles of these miRNAs in the associated diseases, some miRNAs are unique to one disease or a pathogen. For example, some miRNAs could respond to E. coli infections but not to mastitis caused by other pathogens (Table 2). In virus-related diseases, miRNAs are also known to be important for controlling the replication of the virus [205], in which case, they could either impair virus replication (for instance miR-130b [149]) or facilitate virus replication (for instance miR-30c [104]). For bacterial infections, miRNAs can contribute to the host response to the infection via a wide range of pathways and host cells [206]. The miRNAs uniquely associated with a specific disease pathogen or stage of disease pathogenesis are potential targets for the development of biomarkers for prognostic, diagnostic, and therapeutic applications for the management of livestock diseases.

## 4. Challenges and Opportunities for Understanding Biological Roles of miRNA

To date, it is well known that miRNAs play important roles in many biological processes related to disease development in farm animals. Therefore, the application of miRNAs to improve disease resistance in farm animals is very promising. miRNAs can be used as direct biomarkers or indirectly through other technologies. As direct biomarkers, such as circulating biomarkers, miRNA in biological fluids, such as blood, milk, saliva, and urine, can facilitate the rapid detection of disease infection status. Indirectly, miRNA can find use in other technologies, such as RNA interference or genome editing. Genome editing using CRISPR/Cas9 technology can robustly, specifically, and stably modify miRNA expression by editing either the seed sequence of miRNAs or the three prime untranslated regions of their target genes [207]. The success of the application of this technology in miRNA-mediated therapy has been proven in diseases in animal models [208,209,210]. Before adopting miRNAs as biomarkers, it is crucial to understand their roles in disease pathogenesis. Although affordable “OMICS”-based technologies have enabled the faster identification of miRNAs, the identification and validation of miRNA functions is still hindered by low sample size and poor reproducibility. Additionally, a holistic approach for exploring and validating miRNA functions, given the complexity of livestock diseases, is lacking. Since many livestock diseases are chronic in nature, miRNA functional studies should consider the different disease stages. Some diseases are also caused by multiple pathogens, such as mastitis or impact numerous tissues, or organs, such as JD; therefore, the spatiotemporal-specific manner of the regulatory function of miRNAs needs to be considered. The lack of sensitive and reliable tools for detecting lowly expressed miRNAs might ignore some potentially important miRNAs with essential functions. Additionally, an miRNA can target hundreds of genes, thus making it difficult, costly, and labor-intensive to validate each miRNA gene target functionally. Lastly, the limited attention to in vivo experiments for miRNA validation is also a significant challenge for understanding miRNA roles in livestock diseases.

Nevertheless, the lower cost of sequencing may lead to an increase in sample sizes in miRNA studies. Moreover, the present downward trend in the cost of sequencing may provide opportunities to sequence multiple types of molecules (miRNAs, lncRNAs, mRNAs, etc.) simultaneously, thereby enhancing the possibility of integrative analyses for the further exploration of miRNA roles in interaction networks. Furthermore, the collaboration by different research groups can significantly improve the power of detection and validation of miRNA functions. Other technologies, such as single-cell sequencing [211], will further the understanding of disease pathogenesis and miRNA functions, while genome editing [212] and RNA interfering technologies [213] could facilitate the identification of the exact target genes and downstream impact of miRNAs on disease pathogenesis. Machine learning and deep learning methods could improve the ability to classify disease pathogens [214] and predict the roles of miRNAs in disease progression [215].

## 5. Conclusions

It is without a doubt that miRNAs play significant regulatory roles in livestock disease pathogenesis and have substantial potential as biomarkers for the management of livestock diseases. However, the application of miRNAs in disease management is hindered by many factors, such as inadequate diagnostic tools; lack of assessment for the accuracy, sensitivity, and specificity of miRNAs; and potentially high cost of developing miRNA biomarkers. Nevertheless, given the pressing need to control livestock diseases, a significant increase in miRNA research has been observed in recent years. The lower cost of sequencing or miRNA genotyping and more powerful computing resources and statistical methods are important assets for miRNA studies. Therefore, we believe that miRNA biomarkers will eventually be developed and employed as powerful tools to manage livestock diseases.

## Figures and Tables

**Table 1:** Number of detected miRNAs and miRNA-related studies in some farm animal species *.

Species	Precursor miRNA	Mature miRNA	Number of Studies Related to miRNA
Cattle	1064	1025	870
Sheep	106	153	176
Goat	267	436	170
Pig	408	457	798
Chicken	882	1232	621

* Data source: MiRBase release 22 (http://www.mirbase.org/); PubMed databases (22 August 2020) with the keywords “species name + miRNA”.

**Table 2:** Important miRNAs for bovine diseases.

Diseases	Pathogens	Phenotype or Tissue	Changed or Potential miRNA Biomarkers	References
Mastitis	*Streptococcus. uberis*	BMEC ^2^	miR-200c, miR-210, miR-193a, miR-29b-2, miR-130a, miR-98, let-7b, miR-24-2, miR-128-2, let-7d, miR-128-1, let-7e, miR-185, miR-652, miR-494, miR-2342, miR-29c, miR-29e, miR-29b-2, miR-100, miR-130	[56]
		BMEC	miR-181a, miR-16 and miR-31	[57]
		Milk	miR-27b, miR-152, miR-194, miR-200b, miR-222, miR-379 and miR-18397	[58]
		Blood	miR-25, miR30e-5p, miR-342, miR-191, miR-399b, miR-451 and miR-486	[59]
	*Staphylococcus aureus*	BMEC	miR-2339, miR-21-3p, miR-423-5p, miR-499, miR-92a, miR-193a-3p, miR-23a, miR-99b, miR-21-3p, miR-193a-3p, miR-365-3p, miR-30c, and miR-30b-5p	[60]
	*Escherichia coli*	BMEC	miR-184, miR-24-3p, miR-148, miR-486, and let-7a-5p	[60]
	*Escherichia coli*	BMEC	miR-223, miR-16, miR-136, miR-136, miR-3660, miR-335 and miR-378	[61]
	*Escherichiacoli* and *Staphylococcus aureus*	BMEC	miR-144, miR-451 and miR-7863	[62]
	*Streptococcus agalactiae*	Milk	miR-21, miR-146a, miR-155, miR-222, and miR-383	[63]
	CMT ^1^	Milk	let-7i, miR-21, miR-27, miR-99b, miR-146, miR-147, miR-155 and miR-223	[63]
Bovine tuberculosis	*Mycobacterium bovis*	Lung	bta-miR-142-5p, bta-miR-146a and bta-miR-423-3p	[64]
Johne’s disease	*Mycobacterium avium* subsp. paratuberculosis	Blood	mir-19b, mir-19b-2, mir-1271, mir-100, mir-301a, mir-32, mir-6517 and mir-7857	[65]
		Ileum	miR-146 b, miR-196 b, miR-2483–5p, miR-133b, miR-1247-5p, miR-184, miR-202, miR-105a, novel-53, miR-433, miR-2400, miR-137, miR-424–3p and miR-138	[66]
		Serum	miR-1976, miR-873-3p, miR-520f-3p, and miR-126-3p	[67]
		Faeces	miR-223, miR-19b, miR-27b, miR-30d, miR-24 and miR-16	[68]
Diarrhea	Bovine viral diarrhea virus	Serum	miR-423-5p and miR-151-3p	[69]
Foot and Mouth disease	Foot and Mouth disease virus	Serum	miR-17-5p, miR-31 and miR-1281	[70]

^1^ CMT, California mastitis test; ^2^ BMEC, bovine mammary epithelial cells.

**Table 3:** Important miRNAs for pig diseases.

Disease	Pathogens	Tissues/Cells	MIRNAs	References
Porcine reproductive and respiratory syndrome	Porcine reproductive and respiratory syndrome virus	Porcine alveolar macrophages	miR-30a-3p, miR-132, miR-27b, miR-29b, miR-146a and miR-9-2	[97]
		Blood monocytes and porcine alveolar macrophages	miR-181	[98]
			miR-125b	[99]
			miR-23, miR-378, and miR-505	[100]
		MARC-145 cell	miR-145, miR-127	[101]
		Lung	miR-183, miR-219, miR-28-3p and miR-143-3p	[102]
		Lung	miR-26	[103]
		Lung	miRNA-30c	[104]
		Lung	miR-22	[105]
		Lung	miR-373	[106]
		Alveolar macrophages	miR-140, miR-92b, miR-545, miR-1306, miR-374b and miR-199b	[107]
		Alveolar macrophages	miR-10a-5p	[108]
		Blood	miR-125b, miR-145-5p	[109]
Swine influenza infection	Influenza A virus	In silico	miR-124a, miR-145	[110]
		Influenza A virus subtype H1N2	miRNAs miR-15a, miR-21, miR-146, miR-206, miR-223 and miR-451	[111]
Multiple diseases	Salmonella species	Whole blood	miR-155	[112]
		Intestines	miR-29a	[113]
	Lawsonia intracellularis		miR-486, miR-500, miR-127, miR-215, miR-194b-5p and miR-122	[114]
	*Escherichia coli* F18		miR-143, let-7f, miR-30e, miR-148a, miR-148b, miR-181a, miR-192, miR-27b, miR-15b, miR-21, miR-215 and miR-152	[115]
		Duodenum	miR-196b, miR-499-5p and miR-218-3p	[116]
	*Trichuris suis*.	Serum	let-7d-3p	[117]
	*Actinobacillus pleuropneumoniae*	Lung	miR-664-5p, miR-451 and miR-15a	[118]
	Porcine cytomegalovirus	Macrophages	miR101, miR-7, miR-128, miR155-5p, miR-196-5p, miR-18a, miR-19b, and miR-24-3p	[119]
	African swine fever virus	Spleen and submandibular lymph node	miR-126-5p, miR-92c, miR-92a, miR-30e-5p miR-500a-5p, miR-125b, miR-451 and miR-125a	[120]
	Influenza A virus	Lung	miR-15a, miR-18a, miR-21, miR-29b, and miR-590-3p	[121]

**Table 4:** Important miRNAs for chicken diseases.

Disease	Pathogen	Tissue	Changed or Potential miRNA Biomarkers	References
Marek’s Disease	Gallid herpesvirus 2	Spleen and liver	miR-221, miR−140, miR−199, miR-181a, miR−146b, miR−146c and miR−26a	[133]
		Spleen	miR-15, miR-456 and let-7i	[134]
		Spleen	miR-21	[133]
		Spleen	miR-26a	[135]
		Spleen and liver	miR-103-3p	[136]
		Spleen and liver	miR-219b	[137]
	Marek’s disease virus	Bursa samples	miR-30a, miR-1662, miR-9-1, miR-9-2, miR-499, miR-193b and miR-1684a	[138]
Avian Leukosis	Avian leukosis virus	Liver	miR-221, miR-222, miR-1456, miR-1704, miR-1777, miR-1790, miR-2127, let-7b, let-7i, miR-125b, miR-375 and miR-458	[139]
		Liver	miR-375	[140]
		Liver	miR-221, miR-193a, miR-193b and miR-125b	[141]
		Liver	miR-221, miR-222,	[142]
		Liver	miR-23b	[143]
		Liver	mir-34b-5p	[144]
		Liver	let-7b and let-7i	[145]
		Chicken embryo fibroblasts	miR-184-3p, miR-146a-3p, miR-146a-5p, miR-3538 and miR-155,	[146]
Bursal disease	Bursal disease virus	DF-1 cells	miR-9	[147]
		DF-1 cells	miR-2127	[148]
		DF-1 cells	miR-130b	[149]
Avian influenza	Avian influenza viruses	Lung and trachea	miR-146, miR-15, and miR-21	[150]
		Lung	miR-34a, miR-122–1, miR-122–2, miR-146a, miR-155, miR-206, miR-1719, miR-1594, miR-1599 and miR-451	[151]
		Embryo fibroblasts	miR-146c, miR-181a, miR-181b, miR-30b, miR-30c, miR-30e, miR-455, miR-1599 and miR-1416	[152]
Chronic respiratory diseases	Mycoplasma gallisepticum	Lung	miR-8 family, miR-499 family and miR-17 family	[153]
		Cell (DF-1)	miR-99a	[154]
		Cell (DF-1)	miR-101-3p	[155]
		Chicken embryonic lungs and DF-1 cells,	miR-19a	[156]

**Table 5:** miRNAs with important roles in small ruminant diseases.

Species	Disease	Pathogen	Tissue	miRNA	References
Sheep	Cystic echinococcosis	*Echinococcus granulosus*	Intestine	miR-21-3p, miR-542-5p, miR-671, miR-134-5p, miR-26b and miR-27a	[189]
Sheep and goat	Enzootic nasal adenocarcinoma	Enzootic nasal tumor virus	Tumor and para-carcinoma nasal	miR-449b-3p, miR-449a-3p, miR-133a-3p, miR-449c, miR-133b, miR-9-5p,mi miR-148a-3p, miR-296-3p, miR-873-3p miR-331-3p	[190]
Sheep	Bluetongue virus infection	Bluetongue virus	Testis	let-7d, let-7f, miR-106b, miR-10a, miR-10b, miR-136, miR-148a, miR-17-5p, miR-191, miR-194, miR-29a, miR-29b, miR-30a-3p, miR-30b, miR-362, miR-369-3p, miR-369-5p, miR-379-5p, miR-3958-3p, miR-409-3p, miR-412-3p, miR-432, miR-493-5p, miR-541-5p and miR-758-3p	[191]
Sheep	Peste des petits ruminants	Peste des petits ruminants virus	Spleen and lung	miR-21-3p, miR-1246, miR-27a-5p, miR-760-3p, miR-320a and miR-363	[192]
Sheep	Prion diseases	Prion virus	Plasma	miR-342-3p, let-7b and miR-21-5p	[193]
Sheep	Peste des petits ruminant disease	Peste des petits ruminants virus	Peripheral blood lymphocyte	miR-150, miR-370-3p and miR-411b-3p	[194]
Sheep	Lung infection	Small Ruminant Lentiviruses	Lung	miR-21, miR-148a, let-7f, let-7b, miR-99a, and miR-125b	[195]
Goat	Peste des petits ruminants virus infection	Peste des petits ruminants virus	Peripheral blood mononuclear cells	miR-204-3p, miR-338-3p, miR-30b-3p, miR-199a-5p, miR-199a-3p and miR-1	[196]
Goat	Peste des petits ruminants virus infection	Peste des petits ruminants virus	Peripheral blood mononuclear cells	miR-218 and miR-1	[197,198]

## Data Availability

Not applicable.

## Abbreviations

AAAV	Avian Adeno-Associated Virus
AIVs	Avian Influenza Viruses
A ALV	Avian Leukosis Virus
ALV-J	Avian Leukosis Virus Subgroup J
ASF	African Swine Fever
*BCL-2*	B-Cell Lymphoma 2
BMECs	bovine mammary epithelial cells
BVD	Bovine Viral Diarrhea
BVD	Bovine viral diarrhea
BVDV	BVD virus
CCNE1	Cyclin E
CLCF1	Cardiotrophin Like Cytokine Factor 1
CMT	California mastitis test
DE	differentially expressed
DIAP1	*Drosophila* Inhibitor Of Apoptosis Protein 1
*DLG5*	Discs Large MAGUK Scaffold Protein 5
DOAJ	Directory Of Open Access Journals
ELK1	ETS-Like Gene 1
ENTV	Enzootic Nasal Tumor Virus
FMD	foot and mouth disease
FMDV	FMD virus
GaHV-2	*Gallid Herpesvirus* 2
hpi	hours post-infection
Hsp70	Heat Shock Protein Family A
IBDV	Infectious Bursal Disease Virus
IC	ileum control
IFN-α	type I interferon
IFN-β	beta interferon
IL7R	Interleukin 7 Receptor
IR	ileum resistant
IRAK1	interleukin-1 receptor-associated kinase 1
IRAK2	Interleukin-1 Receptor-Associated Kinase 2
*IRF1*	Interferon Regulatory Factor 1
IS	ileum susceptible
JAK1	Janus kinase 1
JD	Johne’s disease
*KLF4*	Krüppel-like factor 4
LD	Linear Dichroism
LS	low Salmonella shedding
MAP	*M. avium* subsp. *paratuberculosis*
MAPK	Mitogen-Activated Protein Kinase
*MDA5*	Melanoma Differentiation-Associated Gene 5
MDPI	Multidisciplinary Digital Publishing Institute
MDV	Marek’s Disease Virus
MHC	Major Histocompatibility Complex
miRNA-Seq	miRNA sequencing
ncRNAs	Non-Coding Rnas
*NFAT5*	Nuclear Factor Of Activated T-Cells 5
NFATC4	Nuclear Factor Of Activated T Cells 4
NFIA	nuclear factor IA
NF-kappaB	nuclear factor-kappa B
NGS	Next-Generation Sequencing
*NPEPPS*	Aminopeptidase Puromycin Sensitive
*ORF4*	open reading frame 4
P.I.	Post-Infection
PCR	Polymerase Chain Reaction
PCV2	Porcine circovirus type 2
*PDCD4*	Programmed Cell Death 4 Gene
PID	Post-Infection Day
PPR	Peste Des Petits Ruminants
PPRV	PPR Virus
PRRS	Porcine Reproductive and Respiratory Syndrome
PRRSV	Porcine reproductive and respiratory syndrome virus
PS	persistent Salmonella shedding
REV	Reticuloendotheliosis Virus
RXRB	retinoid X receptor β
*SOCS5*	Suppressor Of Cytokine Signaling 5
SRP14	signal recognition particle 14
TGS	Third-Generation Sequencing
TLA	Three-Letter Acronym
VP1	Virus Protein
YAP1	Targeting and Repressing Yes-Associated Protein 1
